# TFG-maintaining stability of overlooked FANCD2 confers early DNA-damage response

**DOI:** 10.18632/aging.103782

**Published:** 2020-10-24

**Authors:** Chi Ma, Kanani Hokutan, Yihang Shen, Manoj Nepal, Jin-Hee Kim, Jun Zhang, Peiwen Fei

**Affiliations:** 1 University of Hawaii Cancer Center, University of Hawaii, Honolulu, HI 96813, USA; 2 Graduate Program of Molecular Biosciences and Bioengineering, University of Hawaii, Honolulu, HI 96813, USA; 3 Department of Laboratory Medicine and Pathology, Mayo Clinic Foundation, Phoenix, AZ 85054, USA

**Keywords:** FANCD2, TFG, DNA damage response, aging and cancer

## Abstract

Emerging Fanconi Anemia (FA) signaling in the field of cancer research annotates the extreme importance of its center player, Fanconi Anemia complementation group D2 (FANCD2) in protecting human cells from going awry. However, a previously-unrecognized form of FANCD2, namely FANCD2-V2, is understudied. We report TRK-Fused Gene (TFG) is critical for roles played by FANCD2-V2 in early responses to DNA damage, but not for FANCD2-V1, the long-known form of FANCD2. FANCD2-V2 forms nuclear foci upon DNA damage, and both its focus appearance and disappearance are earlier than FANCD2-V1. The amino acid/aa 5-100 of TFG and the aa1437-1442 of FANCD2-V2 were identified to contribute to their interaction, which maintains the steady-state level of FANCD2-V2 protein. TFGΔaa5-100 or FANCD2-V2Δaa1437-1442-carrying cells could not show timely focus formation of FANCD2-V2 upon DNA damage and gained carcinogenicity over time. This study provides a previously-unknown key to unlock in-depth insights into maintaining genome stability, fostering translational studies on preventing, diagnosing and/or treating related diseases.

## INTRODUCTION

Fanconi Anemia (FA) is a rare multigenic syndrome featuring clinical complications, including an early onset of aging, severe bone marrow failure and an extremely high incidence of cancer [1–3]. To date, there are at least 22 FA gene-encoded products identified to act in concert to prohibit genome instability, resulting in an ever-growing signaling network, namely, FA-ATR signaling [2]. Within this signaling network, the central player FANCD2 orchestrates the signal transduction among ATM, ATR, BRCA1/2 and many others known in governing genome integrity.

As a typical outcome for FA cells, the inability of FANCD2 to be monoubiquitinated or form nuclear foci appears to be a common molecular defect in response to a variety of genotoxic stresses. FANCD2 monoubiquitination or focus formation thus attracts many to study stress responses in order for an in-depth understanding of genome stability. By serendipity, we found an overlooked form of FANCD2, FANCD2-V2. Its expression is relatively higher in normal or non-malignant cells/tissues than matched malignant cells/tissues; vice versa the expression level of the long-known form of FANCD2, FANCD2-V1, is relatively lower in normal or non-malignant cells/tissues as compared to matched malignant cells/tissues [4]. We thus hypothesized that in contrast to FANCD2-V1, FANCD2-V2 may act as a more potent tumor suppressor that contributes to the tumor suppression-activity of FA-ATR signaling, through executing a variety of cellular processes including DNA damage responses (DDR). Indeed, FANCD2-V2’s responses to DNA damage and TRK-Fused Gene (TFG) [5] specific regulation on FANCD2-V2 have tested this hypothesis, paving a previously unrecognized path towards a full grasp of mammalian genome integrity.

## RESULTS

### FANCD2-V2 is an earlier responder than FANCD2-V1 upon DNA damage

**Figure 1:**
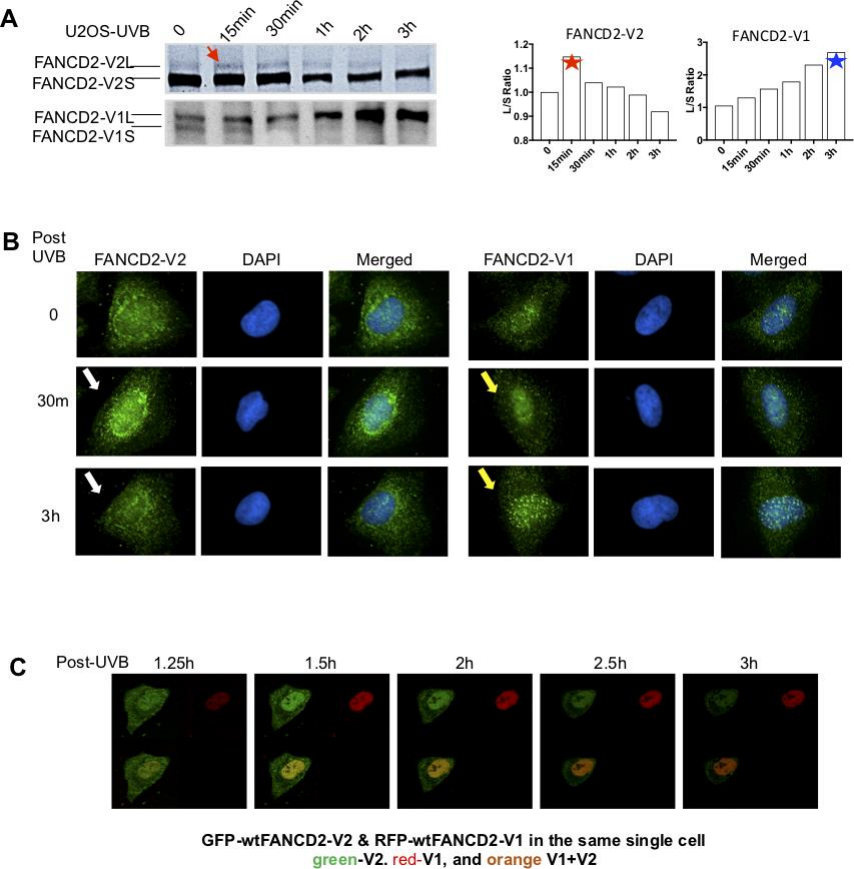
**The peak activation of FANCD2-V2 is earlier than FANCD2-V1 in cells treated with UVB.** (**A**) The peak level of monoubiquitinated FANCD2-V2 showed at an earlier time point comparing to FANCD2-V2. U2OS cells were treated with UVB (25J/m^2^) and collected at the time points indicated in the Figure. Nuclear extractions were subsequently prepared for western blotting of FANCD2-V1 or-V2 proteins through using specific antibodies recognizing V1 or V2 respectively. The relative ratio L/S of monoubiquitinated FANCD2 (L-form) over non-monoubiquitinated FANCD2 (S-form) was shown in the right to indicate different kinetics between the two forms of FANCD2 activation (the ratios of FANCD2-V2 L/S were normalized by the ratio at time 0, considered as 1). (**B**) Focus formation of FANCD2-V2 is earlier than FANCD2-V1. U2OS cells were treated by UVB (25J/m^2^) and collected after 30min or 3h, then together with untreated cells were prepared for immunofluorescent studies. Anti-FANCD2-V1 or anti-FANCD2-V1 specific antibodies were used for the primary incubation. The anti-Rabbit-Alexa 488 (green) was used for the secondary incubation. DAPI was used for the nuclear stain. Focus formation of FANCD2-V2 can be shown clearly at 30min after UVB-treatment and vanished at 3h, but FANCD2-V1 foci were not clearly shown until 3h tested. (**C**) The peak intensity of FANCD2-V2 in the nucleus of a live cell is earlier than FANCD2-V1. The live imaging on cells transfected with both GFP-FANCD2-V2 and RFP-FANCD2-V1 was conducted by taking photos every 30min post UVB-treatment (25J/m^2^). Owing to the time needed to set up, the earliest image was only available at time 1h 15min post treatment (Supplementary Video 1). The earlier fluorescence peak was further supported by the relative cell fluorescence (Supplementary Figure 1C, the bottom panel), which shows green-fluorescence was dominantly shown in cell nucleus in the early time points in UV-treated cells.

To differentiate between two versions of FANCD2 proteins in response to DNA damage, we detected the monoubiquitinated/activated FANCD2-V1 or -V2 in U2OS cells treated with ultraviolet-B (UVB) using specific antibodies raised against the carboxyl terminal unique region of FANCD2-V1 or V2 accordingly (Supplementary Figure 1A and 1B). As shown in Figure 1A, the monoubiquitinated FANCD2-V2 peaks at 15 min, in contrast, the monoubiquitinated FANCD2-V1 peaks up at the last time point tested (3h). The earlier peak-activation of FANCD2-V2 comparing to FANCD2-V1 in UVB-treated cells was reconfirmed in a non-malignant cell line, HEK293 (Supplementary Figure 1C, top) and by quantifying red and green florescence intensities (Supplementary Figure 1C, bottom). The monoubiquitinated FANCD2 can also be visualized in foci, upon which an immunofluorescent experiment (IF) was performed (Figure 1B). FANCD2-V2 foci (the peak intensity of nuclear location) were observed at 30min after UVB treatment, much earlier than FANCD2-V1, and vanished at the time point of 3h post UVB-treatment. However, FANCD2-V1 foci were only observable at 3h. Further, we constructed fusion proteins of FANCD2-V1 and -V2 with fluorescent proteins, RFP and GFP, respectively to better view the dynamics of FANCD2-V1 and V2’s responses upon DNA damage in vivo. We co-transfected GFP-FANCD2-V2 and RFP-FANCD2-V1 into U2OS cells. The next day, these transfected cells were exposed to UVB and immediately subjected to live imaging at 1h post UVB-radiation up to 10h. We found that the peak focus formation of GFP- FANCD2-V2 was earlier than RFP- FANCD2-V1. In addition, at the time point of the RFP focus peak, the intensity of GFP foci was rapidly weakening and subsequently vanished (Figure 1C, and Supplementary Video 1). These observations indicate that in response to DNA damage, FANCD2-V2 acts earlier than FANCD2-V1 and thus plays a crucial role in the timely care of genome stability.

### TFG specifically interacts with FANCD2-V2, but not FANCD2-V1

**Figure 2:**
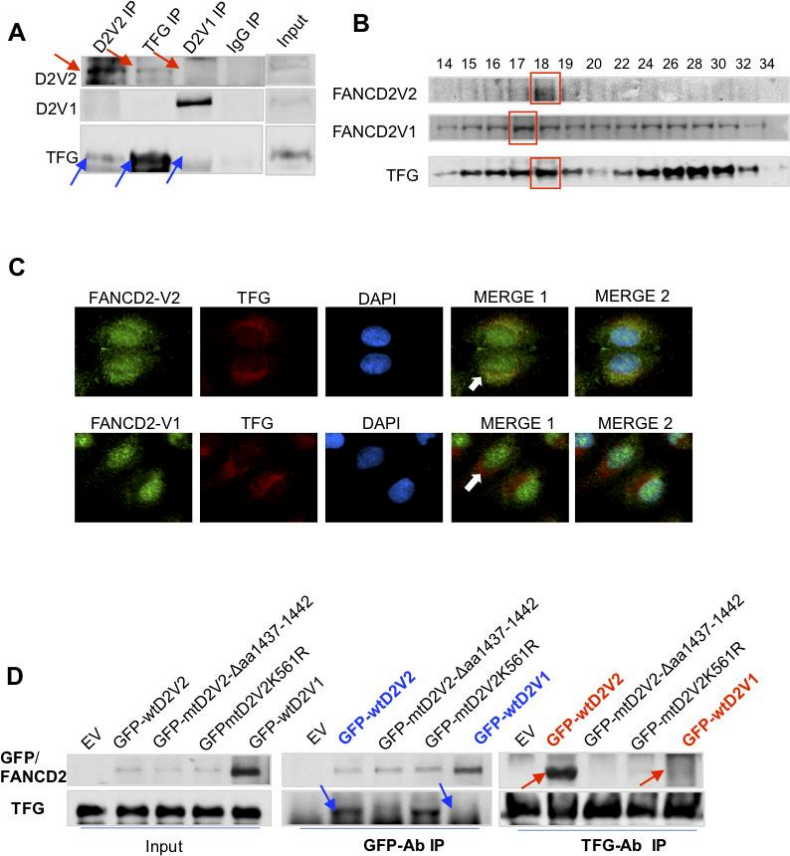
**TFG interacts with FANCD2-V2, but not FANCD2-V1.** (**A**) TFG interacts with the endogenous FANCD2-V2, but not FANCD2-V1. Reverse immunoprecipitation (IP) and western blotting (WB) of endogenous FANCD2-V2, FANCD2-V1 and TFG Rabbit IgG were performed using lysates prepared from U2OS cells. TFG signaling can be clearly detected in the pulldown by antibodies recognizing FANCD2-V2 but not FANCD2-V1. (**B**) TFG is co-peaked with FANCD2-V2 but not with FANCD2-V1. Gel filtration was performed using the total cell lysates prepared from U2OS cells. Western blotting of FANCD2-V1, -V2 or TFG showed that TFG and FANCD2-V2 were peaked at the same fraction, but FANCD2-V1’s peak was a fraction late. (**C**) Fluorescent co-localization of TFG with FANCD2-V2, not with FANCD2-V1. Immunofluorescent study was performed on normally growing U2OS cells. The fixed cells were primarily blocked with antibodies targeting FANCD2-V2, FANCD2-V1, or TFG. Subsequently, anti-Rabbit-Alexa 488 and anti-mouse-Alexa 568 were applied (DAPI was used for nuclear staining. Green fluorescence of FANCD2-V2, but not FANCD2-V1, can be merged with the red florescence of TFG (the orange color). The association of TFG with FANCD2-V2 was also supported by Pearson’s or Mander’s colocalization coefficient with an R nearly 1 (0.96 or 0.98). (**D**) The association of FANCD2-V2 with TFG is attributed to its unique carboxyl terminal. HEK293T cells were transfected with empty vectors, GFP-wtFANCD2-V2, GFP-mtFANCD2-V2Δaa1437-1442, GFP-mtFANCD2 K561R or GFP-wtFANCD2-V1. Antibodies targeting GFP or endogenous TFG were used for reverse IP and WB. TFG can pull down GFP-wtFANCD2-V2, point mutant of FANCD2-V2K561R, but not the carboxyl terminal deletion mutant of FANCD2-V2, either GFP-wtFANCD2-V1 (IgG negative control was performed simultaneously-Supplementary Figure 2D).

In looking for factors responsible for roles played by FANCD2-V2, we picked up TFG protein upon the highest number of peptides found in immunoprecipitated pellets with FANCD2-V2 specific antibodies (Supplementary Figure 2A). Indeed, TFG was found to interact with FANCD2-V2, but not with FANCD2-V1, verified by reverse IP-WB and gel-filtration studies on endogenous TFG and FANCD2-V2 proteins (Figure 2A, 2B). Notably, florescent-compartmentation of each protein further supported RFP-TFG’s interaction more with GFP-FANCD2-V2, but not much with GFP-FANCD2-V1 (Figure 2C). This is because GFP-FANCD2-V2 (showing in both cytoplasmic and nuclear compartments) co-localized well with RFP-TFG in cytoplasm. As known, TFG appears to be a vehicle protein involving protein-shuttling between cellular organelles [6, 7]. To this end, a putative myristoylation site (AA1437-1442: GTDGCI) at the carboxyl terminal of FANCD2-V2 (Supplementary Figure 2B) caught our interest. This site presumably confers numerous cellular effects, including influencing protein–protein interactions, enhancing interactions of a protein with an organelle or plasma membranes for its traffic and/or leading to changes in protein stability [8, 9]. Together, we suspected the specific interaction of TFG with FANCD2-V2, not with FANCD2-V1, may be mediated through the given putative region. We made a deletion mutant (Δaa1437-1442) of FANCD2-V2 (Supplementary Figure 2C) to test if the subject region is responsible for the interaction with TFG. As shown in Figure 2D, mtFANCD2-V2 Δaa1437-1442 failed to associate with TFG compared to wtFANCD2-V2. Interestingly, the mutant FANCD2-V2 K561R remained to interact with TFG, further supporting the carboxyl terminal of FANCD2-V2 is critical for the interaction between TFG and FANCD2-V2, not the known region of K561 shared by both forms of FANCD2, which has been documented to be crucial for their activation/monoubiquitination. These results indicate that the particular region of FANCD2-V2, aa1437-1442, contributes to its interaction with TFG.

**Figure 3:**
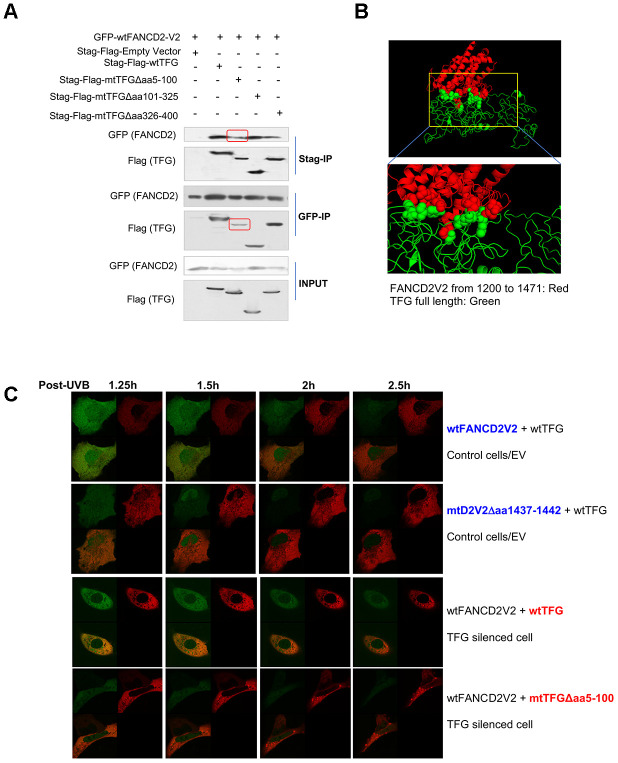
**AA 5-100 of TFG contributes to the interaction between TFG and FANCD2-V2.** (**A**) Aminol terminal of TFG confers its association with FANCD2-V2. HEK293T cells were transfected with Stag-Flag-wtTFG, Stag-Flag-mtTFGΔaa5-100, Stag-Flag-mtTFGΔaa101-325, Stag-Flag-mtTFGΔaa326-400 or empty vector together with GFP-wtFANCD2V2. Both GFP and Stag antibodies’ IPs were performed and the pulldowns were detected by GFP and Flag antibodies. Red line-squares indicate the reduced interaction between GFP-wtFANCD2 and Stag-Flag-mtTFGΔaa5-100. (**B**) Docking of C-terminal of FANCD2-V2 and N-terminal of TFG supports their interaction. The predicted structure of human TFG (green) was adapted well to that of partial FANCD2-V1 protein from 1200-1441 (red). (**C**) Both the aa1437-1442 of FANCD2-V2 and the aa5-100 of TFG are important for the earlier action of FANCD2-V2 upon DNA damage. Live imaging was performed on TFG-normally expressed U2OS cells co-transfected with GFP-wtFANCD2-V2 and RFP-wtTFG or GFP-mtFANCD2-V2(Δaa1437-1442) and RFP-wtTFG; together with TFG expression-compromised U2OS cells co-transfected with GFP-wtFANCD2-V2 and RFP-wtTFG or GFP-wtFANCD2-V2 and RFP-mtTFG(Δaa5-100) (Moving images in Supplementary Videos 2–5 respectively). Photos were taken every 30min. Green focus duration and intensity was reduced in cells carrying mtFANCD2-V2Δaa1437-1442 or mtTFGΔaa5-100 comparing to the corresponding controls.

Next, we began to identify which part of TFG is responsible for interacting with FANCD2-V2. As outlined in Supplementary Figure 3A and 3B, TFG was dissected into three parts and corresponding deletion constructs were established and verified by sequencing. After transiently transfecting these different versions of TFG cDNA-containing plasmids in combination with wtFANCD2-V2 respectively into HEK293T cells, the reverse IP-WB was performed by using antibodies against tags, GFP and Stag-Flag respectively fused with FANCD2-V2 or TFG. We found that the interaction was reduced between FANCD2-V2 and a mutant TFG Δaa5-100 compared to other versions of TFG either deleted at aa101-325 or aa326-400 (Figure 3A). We also confirmed the interaction between FANCD2-V2 and TFG by molecular docking (Figure 3B). We utilized I-TASSER server [10] to generate the putative human protein model of TFG and FANCD2-V1 (1200-1441) and conducted the molecular docking between two proteins using Z-DOCK [11]. The predicted structure of human TFG (green), based on the PSCD-region of the cell wall protein pleuralin-1 (PDB: 2NBI_A) using I-TASSER, adapted well to that of partial FANCD2-V1 protein from 1200-1441 (red) (PDB: 3S4W_B). This separated region from AA40 to AA362 of TFG interacted with FANCD2-V1 (AA1281-1470) was described as spheres, consistent with our IP results (Figure 3A). Next, the in-vivo florescent studies were performed to further support the importance of the interaction relying on aa5-100 region of TFG or aa1437-1442 of FANCD2-V2 by showing FANCD2-V2’s focus formation (Figure 3C, Supplementary Videos 2–5). Particularly, live imaging on a single cell expressing a normal level of endogenous TFG, but co-transfected with GFP-wt or mtFANCD2-V2 along with RFP-wtTFG, the green focus duration (nuclear localization) of GFP-mtFANCD2-V2Δaa1437-1442 was substantially reduced compared to GFP-wtFANCD2-V2. In TFG-silenced cells co-transfected with RFP-wt or mtTFG along with GFP-wtFANCD2-V2, the green focus duration of GFP-wtFANCD2-V2 was also reduced in mtTFGΔaa5-100 transfected cells compared to cells transfected with RFP-wtTFG (Figure 3C and Supplementary Videos 2–5; Supplementary Figure 3C). Together, these live images demonstrate the functional importance of aa5-100 of TFG and aa1437-1442 of FANCD2-V2 for the prompt response of FANCD2-V2 to genotoxic stresses, thus the importance of TFG-regulated FANCD2-V2 in genome caring.

### The association between TFG and FANCD2-V2 assures the timely response of FANCD2-V2 to DNA damage, which is attributed to TFG regulation of the steady-state level of FANCD2-V2 protein, but not FANCD2-V1

**Figure 4:**
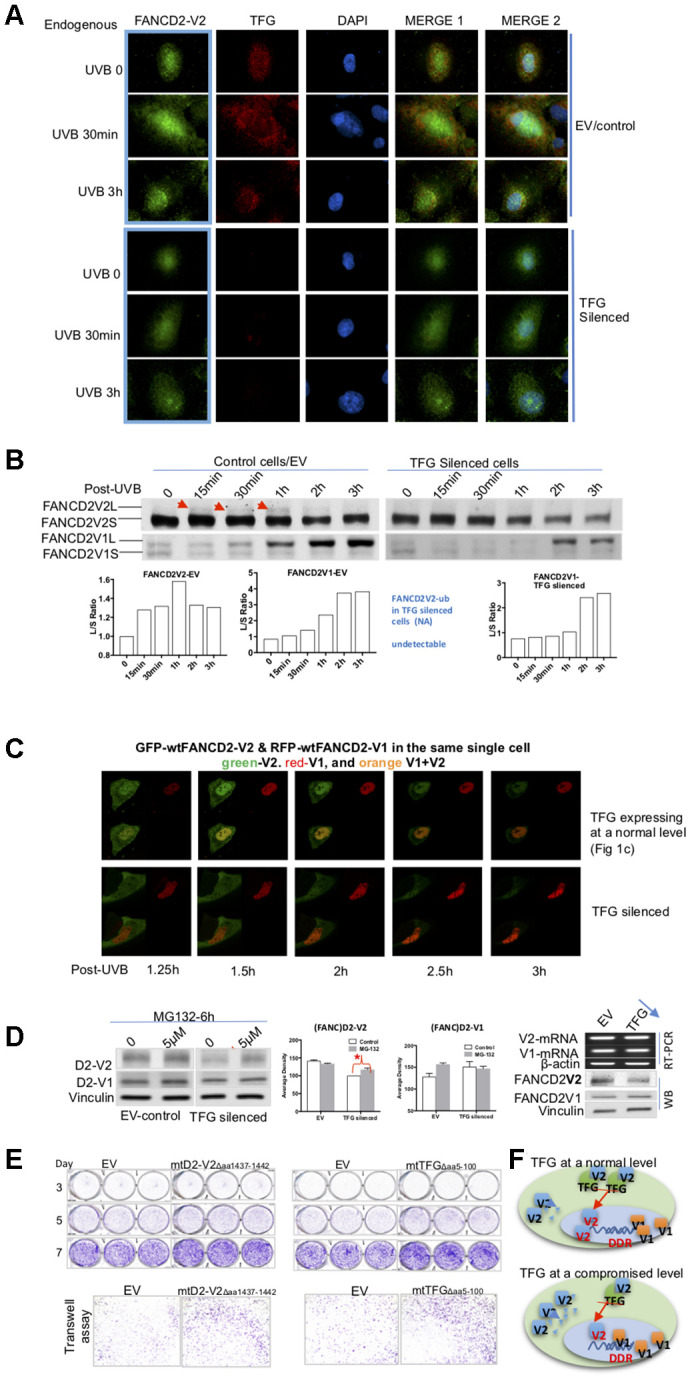
**TFG assists biologic properties of FANCD2-V2 through maintaining its protein steady-state level.** (**A**) TFG maintains the earlier peak concentration of FANCD2-V2 in the stressed cells. Both empty vector control and TFG compromised cells were fixed post UVB treatment for 30min, 3h or untreated for 0 min. Anti-FANCD2-V2 and anti-TFG antibodies were used for primary incubation; and anti-Rabbit-Alexa 488 and anti-mouse-Alexa 568 were used for detection of green (FANCD2-V2) and red (TFG) fluorescence respectively. Green fluorescent intensity is lower in TFG-compromised cells (dim red fluorescence) compared to the cells expressing a normal level of TFG (bright red). Blue fluorescence (DAPI) indicates the nucleus of each individual cell. (**B**) Compromised expression of TFG interferes the peak level of activated FANCD2-V2, but not FANCD2-V1. Nuclear extracts of empty vector control and TFG-silenced cells were analyzed by western blot after UVB treatment (25J/m^2^). Both endogenous FANCD2-V2 and FANCD2-V1 were detected with each specific antibody. The peak level of activated FANCD2-V2 was undetectable or postponed in TFG-expression compromised cells, but not FANCD2-V1 (indicated by red arrowheads and the relative ratio graphs). (**C**) Live images were taken on control and TFG-silenced cells co-transfected with GFP-FANCD2-V2 and RFP-FANCD2-V1, and photos were taken in a 30min interval. Green fluorescent intensity (FANCD2-V2 protein) was low and spread mainly in cytoplasm of TFG-silenced cells comparing to the control cells carrying a normal level of TFG expression. But there is not much difference for RFP-FANCD2-V1 (red fluorescent intensity) between two types of cells (Supplementary Videos 1 and 6). The fluorescence changes were similarly shown in relatively-elevated fluorescence (Supplementary Figure 4D). (**D**) TFG protects FANCD2-V2 from the proteasome-mediated degradation. EV control or TFG silenced cells were treated with 5μM MG132 for 6h. The corresponding whole cell lysates were analyzed by antibodies targeting FANCD2-V2 and FANCD2-V1 respectively. The protein levels of FANCD2-V2 were substantially elevated while blocking proteasome function (MG132 treatment), comparing to FANCD2-V1. Further, TFG modulation of FANCD2-V2 expression does not involve FANCD2-V2 transcription. Both mRNA levels of FANCD2-V1 or V2 are similar between cells carrying a normal or silenced level of TFG expression (through RT-PCR). (**E**) mtTFGΔaa5-100 or mtFANCD2-V2Δaa1437-1442 elevates cell oncogenicity. Trans-well and cell proliferation were performed using cells expressing mtTFG or mtFANCD2. Cells carrying mtTFGΔaa5-100 or mtFANCD2-V2Δaa1437-1442 both showed a similar level of elevation in cell proliferation and cell migration. Statistical calculations were shown in Supplementary Figure 4C. (**F**) Working hypothesis of TFG contributions to early DDR delivered by FANCD2-V2. In cells carrying a normal level of TFG expression, FANCD2-V2 responds to genotoxic stresses earlier than FANCD2-V1 (closer to DNA in drawing). However, in cells carrying a compromised level of TFG expression, a less amount of FANCD2-V2 goes into the nucleus owing to a relatively higher amount of FANCD2-V2 undergoing degradation

Through observing these live images, we noticed that not only FANCD2-V2’s focus duration decreased but also the focus intensity was also dramatically reduced (Figure 3C, Supplementary Videos 3 and 5). To get an in-depth understanding of the protein expression of FANCD2-V2 influenced by TFG, we again used TFG-downregulated cells along with the corresponding control to detect how endogenous TFG affect endogenous FANCD2-V2 upon UVB. IF was performed on UVB-treated cells for 30min or 3h. In addition to the lower level of FANCD2-V2 protein expressed in the nucleus (green fluorescence), an obvious delay of its focus formation was observed in TFG-silenced cells (dim/undetectable red fluorescence) compared to the control (red fluorescence) (Figure 4A). We also confirmed this observation by western blotting using UVB-treated control or TFG-silenced cells. The monoubiquitination of FANCD2-V2 was reduced and too low to be detected in TFG-compromised cells compared to control cells, but not FANCD2-V1 (Figure 4B). Together, these observations indicate that TFG is essential for the early response of FANCD2-V2 to DNA damage, maintaining its timely activation. Next, we wanted to examine if this effect of TFG is also true in live cells. We thus, performed live imaging on both control and TFG silenced cells transfected with GFP-wtFANCD2-V2 and RFP-wtFANCD2-V1. We found that GFP-FANCD2-V2 foci in TFG expression-compromised cells did not last as long as those in control cells, accompanying the weak focus intensity. But there is no observable difference in the duration of RFP-FANCD2-V1 foci (the red fluorescent intensity) and the corresponding focus intensity between two types of cells (Figure 4C and Supplementary Videos 1 and 6), consistent with what observed previously in Figure 4A, 4B; Supplementary Videos 2–5). We realized that changes in the protein level can be a result of many factors, such as the rate of RNA-splicing, protein synthesis and protein degradation [10, 12–14]. We then looked for the possibility of protein degradation first by treating TFG-silenced or control cells with MG132, an inhibitor for proteasome-mediated protein degradation. We found that the basal level of endogenous FANCD2-V2, but not FANCD2-V1, was clearly lower in TFG-silenced cells compared to control cells. The protein level of endogenous FANCD2-V2 in MG132 treated cells, not only went up but also led to a significant difference in referring to untreated cells, as compared to FANCD2-V1 under the same conditions (Figure 4D). To further clarify that the low level of FANCD2-V2 protein was due to degradation, irrelevant to the transcription, we conducted RT-PCR using the same batch of TFG-silenced and control cells. As shown in the right of Figure 4D, similar mRNA levels were detected between TFG-silenced and control cells for both versions of FANCD2 mRNA expressions. However, the protein level of FANCD2-V2 was lower in TFG silenced cells compared to control cells. Therefore, the degradation of FANCD2-V2 was protected by its interaction with TFG, which is very important for specific functions performed by FANCD2-V2, distinct from those of FANCD2-V1, e.g. rapidly forming foci in response to genome toxicants.

### The disrupted association between TFG and FANCD2-V2 confers tumorigenicity

Knowing the importance of FANCD2-V2 in maintaining genome stability, it is imminent to determine functional outcomes of FANCD2-V2’s timely responses to DNA damage and that of its partner TFG in maintaining its prompt response. We, therefore, detected if cell oncogenicity was affected by TFG expression and found that compromised-TFG expression promoted cell invasive capacity (Supplementary Figure 4A), consistent with the abnormal morphology of TFG silenced cells or those transfected with mtTFG (Figure 3C, 4C; Supplementary Video 5 and 6). To further support the interaction between TFG and FANCD2-V2, we decided to study the corresponding biological effects of mtTFG Δaa5-100 or mtFANCD2-V2 Δaa1437-1442. Using stable cell pairs expressing mtTFGΔaa5-100 or mtFANCD2-V2Δaa1437-1442 along with empty-vector controls (Supplementary Figure 4B), we found that cells carrying mtTFG or mtFANCD2-V2 grew faster, migrated more to the other side of the membrane (Figure 4E, Supplementary Figure 4C). These data together indicate that interrupting the interaction between FANCD2-V2 and TFG can eventually elevate the tumorigenic potential that over time promotes tumor development, in agreement with the fact that FANCD2-V2 was downregulated in malignant cells [4]. On the other hand, this interrupted interaction does appear to be beneficial to cancer treatment, as observed reduced cell survival percentages in cells carrying mtFANCD2-V2 or mtTFG compared to the control cells (Supplementary Figure 4D). When cells express a proper amount of TFG, the interaction of TFG with FANCD2-V2 protects FANCD2-V2 from proteasome degradation (Figures 2 and 4D) thus conferring an earlier action of FANCD2-V2 upon DNA damage (Figures 1, 3 and 4A–4C; Supplementary Videos 1–6). If the interaction was compromised by either eliminating the critical region of FANCD2-V2 or TFG, FANCD2-V2 protein would be more exposed for degradation, resulting in a low level of FANCD2-V2 protein and an overtly-low level of protein in the nucleus for earlier response to DNA damage (Figure 4F). Over time, the subject abnormality will lead to insufficient DNA-damage repair, genome instability, and relevant clinic disorders.

## DISCUSSION

The accumulated studies indicate that the FA pathway has become the heart of the DNA-damage repair-signaling network, namely FA signaling or the FA signaling network [1–3]. In this network, four FA-gene products (FANCD1/J/N/S) are previously well-known DNA damage repair proteins (respectively for BRCA1/2, BRIP1 and PALB2), conferring human cancer susceptibility in breast, ovary, prostate, and/or others. Other FA proteins (FANCG/O/Q/R/U/V/W) are also previously known proteins involving DDR (respectively for Rad51, Rad51C, XRCC2/4/9, Rev7 and RFWD3) [1–3, 15, 16]. It has now become clear to us the importance of FA signaling in guarding against genome instability upon genotoxic stresses in general, unrestricted to FA patients [1, 2, 17–21]. This is certainly owing to these functionally important FA proteins in the maintenance of genome stability. Therefore, it is very crucial to study the characteristics of FANCD2, the center player of the FA signaling network that orchestrates the unity of FA signaling in the fight against diseases associated with genome instability, such as aging and cancer.

While studying FANCD2, we noticed a hidden form of FANCD2, FANCD2-V2. Its unique expression pattern [4] prompted us to systematically investigate its functions. The opposite expression pattern of the commonly-known FANCD2/FANCD2-V1 led us to wonder if it was even a part of oncogenesis [4]? In this study, we found FANCD2-V1 is a nuclear protein without detectable expression in the cytoplasm; whereas FANCD2-V2 is cytoplasmic with a clear portion in the nucleus (Figures 1, 2C). The roles of cytoplasmic FANCD2-V2 are currently uncertain. Recent publications [22, 23] indicate FANCD2 involvement in mitochondria function. It is yet to be determined whether FANCD2-V2 is relevant to mitochondria. In terms of their common location, the nucleus, we found FANCD2-V2 acted more expeditiously than FANCD2-V1. This behavior of FANCD2-V2 was found to be attributed to TFG regulation, at least, partly on its steady-state level (Figures 2–4). MG132 blockage of proteasome-mediated protein degradation promoted a substantial elevation of FANCD2-V2, but not for FANCD2-V1 (Figure 4D). This finding not only provides a further mechanistic insight into the timely response of FANCD2-V2 to DNA damage but also potentiates a distinct molecular mechanism from FANCD2-V1 in a relatively-TFG-specific manner.

TFG facilitates transportation from the ER to Golgi intermediate compartments (ERGIC) [6, 7]. This appears to be a housekeeping function, which, if impaired, would affect many newly-synthesized proteins. This is not the scenario we found, because FANCD2-V1 protein and its response to DNA damage exhibit no differences between cells with different levels of TFG expression. We are unclear of whether TFG’s effects on ERGIC are involved; however, what we demonstrated is more non-housekeeping, rather a specific effect on FANCD2-V2. This is consistent with TFG’s role in promoting the pancreatic β cell mass [24]. We think both cases are a specific aspect of TFG’s function, mostly independent of ERGIC. Furthermore, it is plausible for TFG to protect FANCD2-V2 from degradation, thus conferring cells to have rapid responses. On the contrary, diminished FANCD2-V2 or compromised TFG overtime leads to genome instability and carcinogenesis as indicated by their mutant’s tumorigenicity (Figure 4E), further supporting that both share a common signaling path in contributing to and leading to long-range effects on cell fates.

## MATERIALS AND METHODS

### Cell lines and reagents

The U2OS and HEK293 cell lines were obtained from the shared resource center of University of Hawaii Cancer Center (purchased from ATCC). U2OS and 293T cells were maintained in DMEM medium supplemented with 10% fetal bovine serum at 37 °C in 5% CO2 (v/v). Antibodies against FANCD2-V1 or V2 were customarily made by Cocalico Biologicals, Inc. (Stevens, PA). Those recognizing FANCD2, TFG or RFP were obtained from NOVUS (Littleton, CO). The anti-vinculin and GFP antibodies were from Santa Cruz (Dallas, TX). The blue dextran, neomycin and molecular weight markers used for gel filtration were from Sigma (St. Louis, MO). MG-132 was from MP Biomedicals (Santa Ana, CA).

### Cell treatment

As described previously [25], medium of U2OS or 293T cells transfected for 12h were removed and washed with PBS, then the cells were treated with UVB light at 25 J/m^2^ and cultured for the given period.

### Immunoprecipitation and Immunoblotting

Whole cell lysates were prepared in IP lysis buffer with 1% CHAPS, and about 2mg proteins were incubated with anti-FANCD2-V2, D2-V1, TFG, GFP or Flag antibodies overnight at 4°C after pre-clearance. The incubation was continued for another 2-4h after protein A-sepharose beads (Invitrogen, Thermo Fisher Scientific, Waltham, MA) were added. The IP pellets were then washed with IP wash buffer X3 and then boiled in 1 X SDS-lysis buffer for 5 min. The supernatant was analyzed by western blotting.

For immunoblotting, cells were lysed with 1 X SDS lysis buffer, or nuclear fractions were prepared with the NE-PER Nuclear and Cytoplasmic Extraction Reagents Kit (Pierce, Thermo Fisher Scientific, Waltham, MA) following the protocols provided by the manufacturer. Then 30-40 μg of protein lysates were separated on 10% SDS-PAGE and transferred onto nitrocellulose membranes. The membranes were blocked with 5% non-fat milk in PBS containing 0.1% Tween-20 for 1h and probed with corresponding primary Abs and secondary Abs-conjugated with horseradish peroxidase (HRP), and detected by enhanced chemiluminescence (ECL, Thermo-Piers, IL, USA). Vinculin or β-actin was used as a protein loading control.

### Gel filtration

Gel filtration analysis was performed as described previously [26]. Whole cell lysates were prepared in IP lysis buffer with 1% CHAPS and was directly applied to a Sepharose 6B column equilibrated with column running buffer containing 20 mM HEPES (pH 7.9), 100 mM NaCl, 1 mM dithiothreitol (DTT), 0.2 mM phenylmethylsulfonyl fluoride (PMSF), 5 mg/ml leupeptin, 2 mg/ml aprotinin, 0.1% Tween-20 and 10% glycerol. Fractions were collected and analyzed by SDS–PAGE and immunoblotting. 2000 kDa blue dextran and 669 kDa thyroglobulin were used as the size markers of fractions.

### Immunofluorescence and live image

Cells were seeded into dishes and followed by transfection and/or treatment. Cells were fixed with 4% paraformaldehyde at a proper time period and permeabilized with 0.1% Triton X-100. After blocking with 3% goat serum for 1h at RT, these cells were incubated with anti-FANCD2-V2, V1 or TFG antibody at 4°C overnight. After washing with PBS, anti-rabbit secondary antibody conjugated with Alexa Fluor-488 or anti-mouse antibody with Alexa Fluor-568 (Invitrogen, Thermo Fisher Scientific, Waltham, MA) were added and incubated for 1h at RT. Cells were washed and mounted in mounting solution with DAPI (Invitrogen, Thermo Fisher Scientific, Waltham, MA).

For live imaging, U2OS cells were seeded on special glass base dishes and transfected with RFP-FANCD2V1 and GFP-FANCD2V2 for 12h, then treated with UVB at 25 J/m^2^. Cells were then observed with 10X60 lens on a Leica SP5 Confocal Microscope. Images were acquired every 15min for 4-6h and analyzed using Leica Application Suite X.

### Lentivirus mediated TFG-silencing

Lentiviral transduction was performed as described previously [25]. A set of four pLKO.1 lentivirus plasmids were purchased from GE (Dharmacon, Lafayette, CO) to generate corresponding lentiviruses and shRNA targeting TFG 3’UTR was synthesized by IDT (Skokie, Illinois USA). U2OS cells were then infected with these viruses according to the protocol provided. Single colony of infected cells were selected with puromycin 24h post-infection and TFG knock down was verified using western blot. These infected cells were also pool-selected for the use of the mutual validation of the results derived from the pooled-colony cells

### Mutagenesis and deletion constructs

As previously described [18], the full-length TFG was generated by PCR amplification using pcDNA3.1/NT-GFP-TOPO TA Expression Kits (Invitrogen, Thermo Fisher Scientific, Waltham, MA). The deletion-mutations were generated by PCR amplification based on template pcDNA3.1/NT-GFP-TOPO TFG plasmid using pfuUltra II (Agilent Technologies, Santa Clara, CA). The sequences of primers used to generate those mutants were as following: del1_primer: GGATCTAAGTGGGAAGCTAAGACCCCTTGAATCAAGTC; del2_primer: TTTGTTAATGGCCAGCCACAAACTTCTCAGCCTACT; del3_primer: ACAAACTTACACTGCCCAAACTGGACCTGGTTATCGATAA.

### Cell proliferation, invasion and drug-sensitivity assay

In the cell proliferation assay, 1x10^4^ cells/well were seeded to 6-well plates and cultured for 7 days. The plates were fixed and stained with crystal violet solution at day 1, 3, 5 and 7.

According to the manual of BioCoat™ Matrigel Invasion Chamber (Corning, Union City, CA), after rehydration, about 2 x10^4^ cells/well were seeded to a 24- well chambers and cultured at 37 °C in 5% CO2 for 24h. A cotton tipped swab was used to remove non-invading cells, and the inserts were stained with crystal violet solution. Subsequently, the insert was washed with PBS and air-dried.

For the drug sensitivity assay, cells were plated and the next day treated with UVB and incubated with 100 ng/ml mitomycin C (MMC) for 24h. After treatment, plates were stained with crystal violet solution, then washed with PBS and air-dried.

### Fluorescence intensity measurement and merge coefficient calculation

By using Image J, we measured fluorescence intensity and generated the relative red or green florescence, and r-values through Pearson’s or Mander’s colocalization coefficient.

### Statistical analysis

All values were expressed as the mean ± SD of individual samples. Samples were analyzed using Student’s t-test for two groups. A *p*-value <0.05 was considered as statistical significance.

## Supplementary Material

Supplementary Figures

Supplementary Video 1

Supplementary Video 2

Supplementary Video 3

Supplementary Video 4

Supplementary Video 5

Supplementary Video 6
