# Supplementary material for: TFG-maintaining stability of overlooked FANCD2 confers early DNA-damage response
Source: Aging (Albany NY). 2020 Oct 24;12(20):20268–84. doi: 10.18632/aging.103782 (PMC7655164; doi:10.18632/aging.103782)
Supplement: Supplementary Figures [file aging-12-103782-s001..pdf]

SUPPLEMENTARY FIGURES

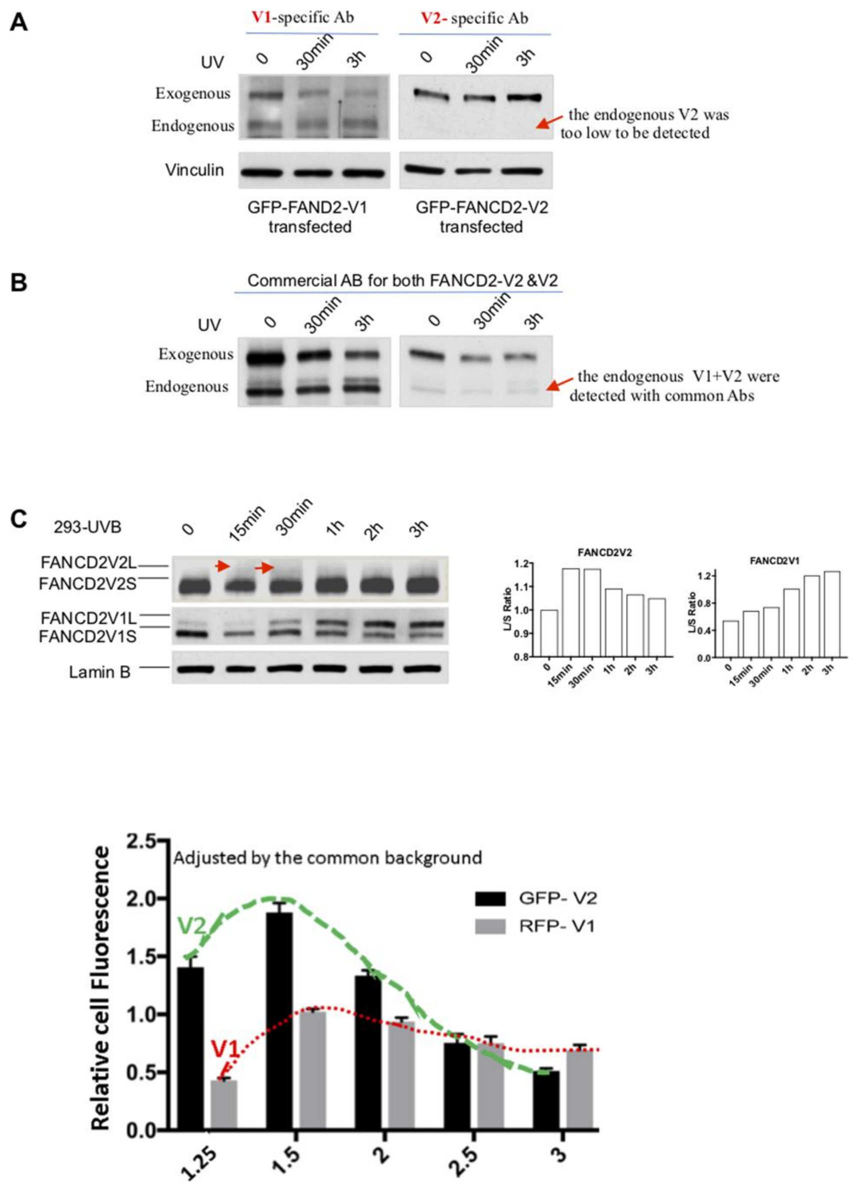

Supplementary Figure 1. The activation of FANCD2-V2 is earlier than FANCD2-V1 in cells exposed to genotoxic agents. (**A**, **B**) Customarily-made antibodies specifically recognizing FANCD2-V1 or V2 show the western blotting patterns of FANCD2 –V1or V2, that are the same as those from the commercial antibodies (NOVUS) targeting the amino terminal of FANCD2 protein. HEK293T cells were transfected with GFP-FANCD2V2 or GFP-FANCD2-V1, then the cell lysates were analyzed by western blotting with anti-FANCD2-V2, FANCD2-V1 antibodies (a) and those (b) commercially targeting both forms of FANCD2 proteins. (**C**) Top: The peak level of monoubiquitinated FANCD2-V2 showed at an earlier time point comparing to FANCD2-V1 HEK293 cells were treated with UVB (25J/m2) and collected at the time points indicated in the figure. The whole cell lysate was subsequently prepared for western blotting of FANCD2-V1 or-V2 proteins through using specific antibodies recognizing V1 or V2 respectively. The relative ratio L/S of monoubiquitinated FANCD2 (L-form) over non-monoubiquitinated FANCD2 (S-form) was shown in the right to indicate different kinetics between the two forms of FANCD2 activation (the ratios of FANCD2-V2 L/S were normalized by the ratio at time 0, considered as 1). (**C**) Bottom: The florescence intensity of the live cell images was measured through Image J and both red and green florescence intensities were adjusted by the same common background florescence and plotted in a relative fluorescence. The green florescence was shown dominantly in the earlier time points detected compared to the red florescence.

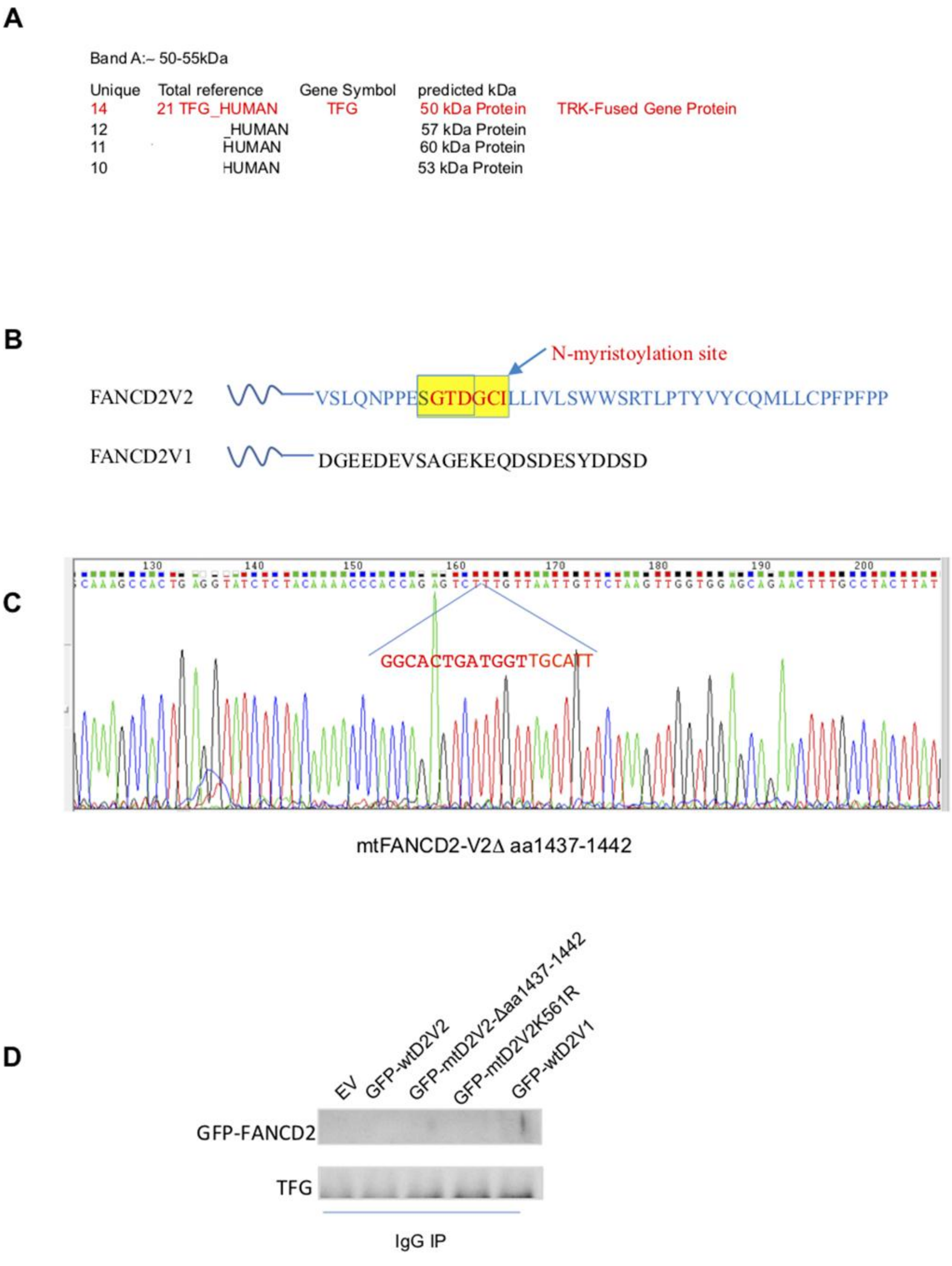

Supplementary Figure 2. TFG was found to be associated with wtFANCD2-V2, but not wtFANCD2-V1 or mtFANCD2-V2 Δ aa1437-1442. (**A**) After immunoprecipitation with anti-FANCD2-V2 or FANCD2-V1 antibodies, the specific protein bands that pull-down by anti-FANCD2-V2 antibodies were sent for mass spectrometric analysis. TFG protein was found to be one of putative partners. (**B**) Illustration of the putative functional motif at the carboxyl terminal of FANCD2-V2. (**C**) mtFANCD2-V2Δ aa 1437-1442 was verified by sequencing. The deletion part was indicated in red characters. (**D**) Negative control for IP performed in Fig 2D. The same amount of IgG was used to perform the corresponding IP shown in the Fig 2D. Both GFP fused FANCD2 and endogenous TFG were not detectable the pulldown derived with non-specific rabbit IgG. The IP and WB were performed at the same time when IPs shown in Fig 2D with the same batch of cell lysates.

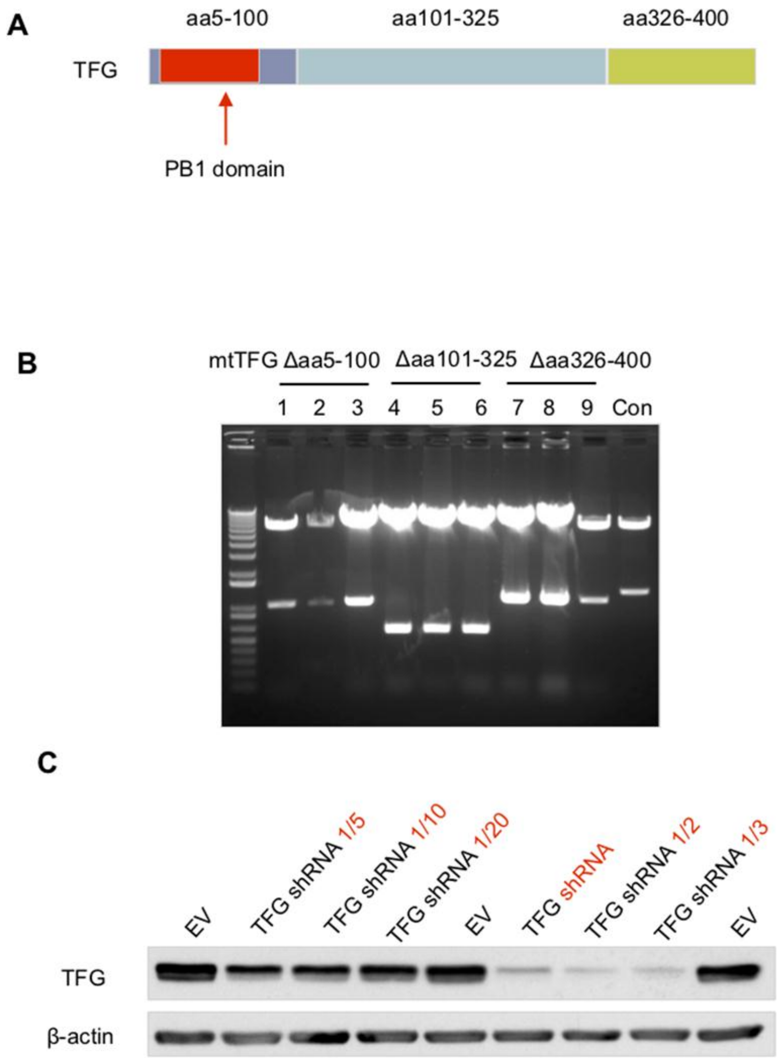

Supplementary Figure 3. Establishment of domain deleted TFG. (**A**) Schematic graph showed three parts of deleted domain of TFG. There is a PB1 domain within the aa5-100 highlighted with red color. (**B**) Domain deleted TFG plasmid were confirmed by restriction enzyme digestion, and sizes were compared to the wild type TFG control plasmid. (**C**) TFG-silenced cells were verified by western blotting. The compromised TFG expressing cells were pooled for the experimental use. The U2OS cells were infected with different concentrations of lenti-virus expressing TFG-shRNA targeting the 3'UTR of *TFG* gene. Then cells were selected by puromycin and analyzed by western blotting.

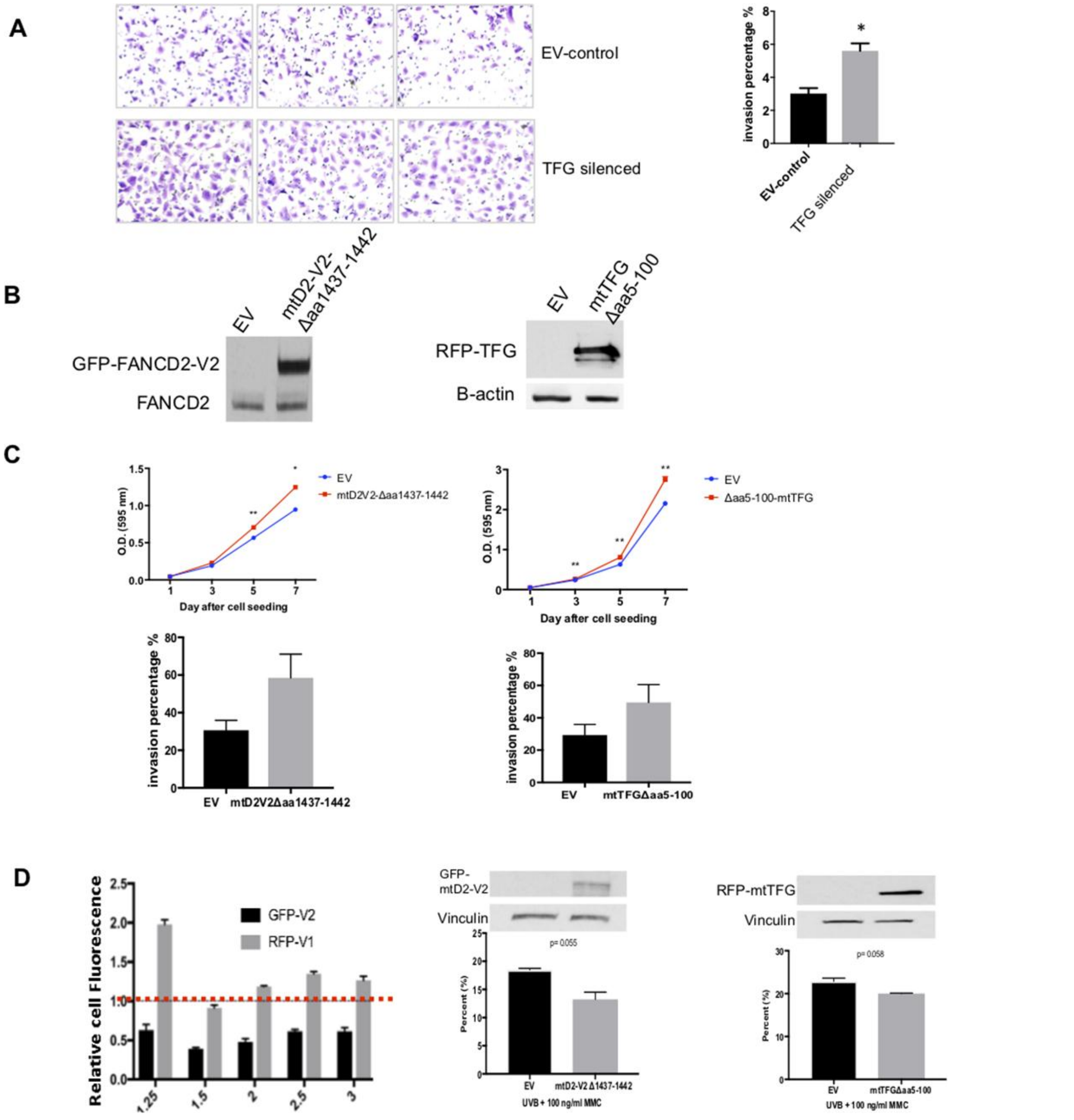

Supplementary Figure 4. Further characterization of the interaction between TFG and FANCD2-V2. (**A**) Compromised TFG expression elevates cell oncogenicity. Trans-well assay was performed using cells expressing different levels of TFG to test their capacity of migration. TFG-silenced cells showed stronger capacity of migration comparing to control cells. (**B**) Verification of cells stably expressing mtTFGΔaa5-100 TFG or mtFANCD2-V2Δaa1437-1442. Western blotting analysis was performed on pool-selected cells that overexpressing mtTFGΔaa5-100 TFG (right) or mtFANCD2-V2Δaa1437-1442 (left). (**C**) Statistical analysis of images shown in Fig. 4E for the oncogenicity of U2OS derivative cells. Cells carrying mtTFGΔaa5-100 TFG or mtFANCD2-V2Δaa1437-1442 gained oncogenic potential compared to the corresponding control cells transfected with empty vector. (**D**) Left: The Red or Green fluorescence measurement in TFG-silenced or normal expressed cells. Green fluorescence was dramatically reduced in TFF-silenced cells compared to the corresponding control cells (ratio<1). Red fluorescence was not reduced, but elevated to some degree. Therefore, TFG-silencing can reduce FANCD2-V2, but not FANCD2-V1, in the nucleus. (S4D). Right: The interrupted interaction between FANCD2-V2 and TFG confers cell sensitivity. U2OS derivative cells expressing empty vector for control, mtFANCD2-V2, or mtTFG were treated by 25J/m2 UVB and followed by 100ng MMC. Three days later these cells were collected for the comparison of growing cell numbers. The number of cells expressing mtFANCD2-V2 or mtTFG were consistently reduced compared to the corresponding control cells.
